# Dynamic properties of mitochondria during human corticogenesis

**DOI:** 10.1242/dev.194183

**Published:** 2021-02-19

**Authors:** Tierney Baum, Vivian Gama

**Affiliations:** 1 Department of Cell and Developmental Biology, Vanderbilt University, Nashville, TN 37232, USA; 2 Vanderbilt Center for Stem Cell Biology, Vanderbilt University, Nashville, TN 37232, USA; 3 Vanderbilt Brain Institute, Vanderbilt University, Nashville, TN 37232, USA; 4 Vanderbilt Ingram Cancer Center, Vanderbilt University, Nashville, TN 37232, USA

**Keywords:** Cortical development, Mitochondrial dynamics, Mitochondrial motility, Mitophagy

## Abstract

Mitochondria are signaling hubs responsible for the generation of energy through oxidative phosphorylation, the production of key metabolites that serve the bioenergetic and biosynthetic needs of the cell, calcium (Ca^2+^) buffering and the initiation/execution of apoptosis. The ability of mitochondria to coordinate this myriad of functions is achieved through the exquisite regulation of fundamental dynamic properties, including remodeling of the mitochondrial network via fission and fusion, motility and mitophagy. In this Review, we summarize the current understanding of the mechanisms by which these dynamic properties of the mitochondria support mitochondrial function, review their impact on human cortical development and highlight areas in need of further research.

## Abstract (precis)

**Summary:** This Review highlights the mechanisms by which the dynamic properties of the mitochondria impact human cortical development.

## Introduction

Modeling the processes of cortical development is essential for understanding the specific molecular mechanisms involved in human brain development and for elucidating the etiology of many neurological diseases. While the importance of the mitochondria during brain development has long been known, an emerging view emphasizes that mitochondrial homeostasis is tightly regulated during cortical development, and more importantly that mitochondrial morphology and function underlie many of the physiological processes that take place during this fundamental time in human growth.

Found in nearly every cell type in the human body, mitochondria are vital for cellular respiration through oxidative phosphorylation (OXPHOS), metabolite synthesis, calcium (Ca^2+^) buffering and apoptosis. Mitochondrial homeostasis is maintained through the concerted execution of mitochondrial dynamics (fusion and fission), cristae dynamics, motility and mitophagy (Barnhart, 2016; Chen and Dorn, 2013; Cogliati et al., 2013; Frank et al., 2001; Giacomello et al., 2020; Schwarz, 2013; Scorrano et al., 2002; Ziviani et al., 2010). As evidence of the importance of these dynamic properties in the modulation of brain development, rare mutations in proteins involved in their regulation cause phenotypically heterogeneous and severe neurodevelopmental diseases (Table 1) (Amati-Bonneau et al., 2008; Assia Batzir et al., 2019; Bartsakoulia et al., 2018; Benincá et al., 2020; Fahrner et al., 2016; Gerber et al., 2017; Gödiker et al., 2018; Hogarth et al., 2018; Koch et al., 2016; Ladds et al., 2018; Mei et al., 2019; Nasca et al., 2018; Panda et al., 2020; Ryan et al., 2018; Schmid et al., 2019; Shamseldin et al., 2012; Sheffer et al., 2016; Shimizu et al., 2003; Tarailo-Graovac et al., 2019; Vanstone et al., 2016; Verrigni et al., 2019; von Spiczak et al., 2017; Waterham et al., 2007; Whitley et al., 2018; Zeharia et al., 2016). Emerging studies have highlighted the crosstalk between the mitochondrial dynamics machinery, the mitochondrial contact site and the cristae organizing system (MICOS), as well as motility and mitophagy machinery at the mitochondria (Kageyama et al., 2014; Morciano et al., 2016; Fu et al., 2019; Picard et al., 2016). However, the molecular details of such signaling crosstalk and their impact on processes in neural differentiation and maturation are only beginning to be understood.

**Table 1.**
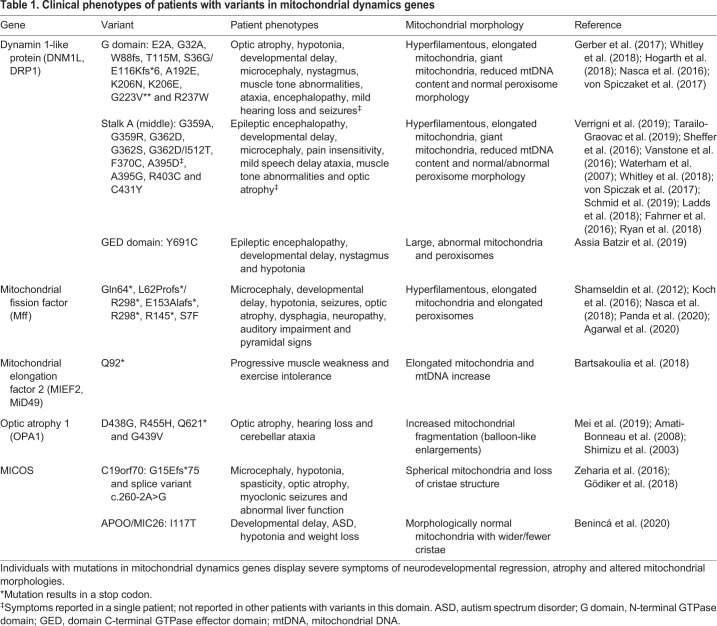
**Clinical phenotypes of patients with variants in mitochondrial dynamics genes**

The mitochondrial network is highly adaptive to the variable energetic needs of different cell types: carefully controlling dynamics machinery to switch between glycolytic and OXPHOS states as cells differentiate and mature. Efforts to create a comprehensive, region-specific mitochondrial profile throughout development are increasing, but there is still work needed to understand the state of the mitochondria during typical brain development, and during disease. As individuals with mutations in mitochondrial dynamics genes display severe symptoms of neurodevelopmental regression, atrophy and altered mitochondrial morphologies, leveraging rapidly emerging technologies using human induced pluripotent stem cells (iPSCs) and human embryonic stem cells (hESCs) to model the earliest stages of development and disease may be a powerful tool with which to gain insight into these complex pathways (Arlotta and Paşca, 2019; Camp et al., 2015; Menacho and Prigione, 2020; Robertson et al., 2020; Romero-Morales et al., 2020). In this Review, we highlight how the dynamic properties of the mitochondria control human cortical development, emphasizing areas of research opportunity and therapeutic need.

## An overview of the development of the human cortex

**Fig. 1.**
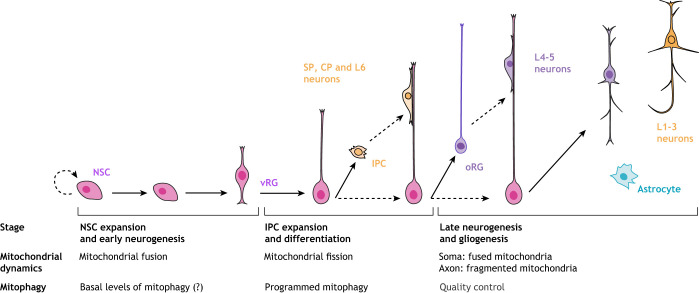
**Mitochondrial dynamics through stages of neurogenesis.** Illustration of neurogenesis in the mammalian cortex (based on work by Molnár et al., 2019). Early neural differentiation is accompanied by a shift from a fused mitochondrial network to more fragmented mitochondria that support changes in bioenergetic function from oxidative phosphorylation (OXPHOS) to glycolysis and increased motility. During late neurogenesis, these dynamics changes become compartmentally specified with more fused mitochondria existing perinuclearly in the soma and fragmented mitochondria available for travel to distal axons. The details of the pathways of mitophagy in cortical development are still largely unknown; however, programmed mitophagy is essential during early neurogenesis and quality control based mitophagy is inferred to be crucial for maturing neurons. CP, cortical plate; IPC, intermediate progenitor cell; L, layer ; NSC, neural stem cell; oRG, outer radial glia; SP, subplate; vRG, ventral radial glia.

The cerebral cortex is the center responsible for crucial human abilities such as perception, thought, language, attention, episodic memory and voluntary movement (Molnar and Pollen, 2014). Each of these complex processes require fine-tuning of the energetic capabilities of stem cells, progenitors and mature neurons within this area of brain, as well as of migrating cells from nearby regions. In fact, mitochondrial diseases, which are linked to mutations that impair OXPHOS, affect about 1 in 5000 live births (Schaefer et al., 2004). In addition, perturbations in genes involved in the maintenance of mitochondrial morphology and cristae dynamics also have devastating effects on human brain development (Table 1) (Amati-Bonneau et al., 2008; Assia Batzir et al., 2019; Bartsakoulia et al., 2018; Benincá et al., 2020; Fahrner et al., 2016; Gerber et al., 2017; Gödiker et al., 2018; Hogarth et al., 2018; Koch et al., 2016; Ladds et al., 2018; Mei et al., 2019; Nasca et al., 2018; Panda et al., 2020; Ryan et al., 2018; Schmid et al., 2019; Shamseldin et al., 2012; Sheffer et al., 2016; Shimizu et al., 2003; Tarailo-Graovac et al., 2019; Vanstone et al., 2016; Verrigni et al., 2019; von Spiczak et al., 2017; Waterham et al., 2007; Whitley et al., 2018; Zeharia et al., 2016). Although animal models have been pivotal for elucidating some phenotypes associated with dysfunctional mitochondria, human psychiatric and neurological conditions have developmental origins that cannot be fully understood using animal models (Molnár et al., 2019; O'Rahilly and Müller, 2008). For example, compared with mice, the human cortex has relative enlargement of the upper layers, enhanced diversity and function of inhibitory interneurons, and a highly expanded transient subplate layer during development (Molnár et al., 2019). The frontal and parietal areas of the cortex are also highly developed in humans (Fuster, 2002; Kaas, 2008). Pyramidal neurons in layer III of the cerebral cortex form significant numbers of connections with other cortical areas (Barbas, 2007; Yeterian et al., 2012). In addition to this cortical expansion and increased connectivity, the cortical circuitry is also increasingly complex in the human brain (Burkhalter, 2008; Forbes and Grafman, 2010; Heide et al., 2020). How alterations in mitochondrial dynamics, morphology and metabolism directly contribute to disease has remained unclear. The complexity of human cortical development comprises three fundamental cellular events (Fig. 1): neurogenesis and diversification of radial glia subtypes; intermediate progenitor cell expansion and dfferentiation; and expansion and maturation of cortical layers.

### Neurogenesis and diversification of radial glia subtypes

In humans, cortical neurogenesis starts at gestational week (GW) 5 and ends around GW20 (Bystron et al., 2008). The remarkable diversity of cell types that emerge during mammalian corticogenesis largely originate from two main germinal zones, which are located in the ventral and dorsal telencephalon (Arlotta and Paşca, 2019; Lodato and Arlotta, 2015; Silbereis et al., 2016). Neocortical neurogenesis begins when neuroepithelial cells (also known as neural stem cells, NSCs) lining the ventricles start to generate nascent neurons and gradually transform into committed neural progenitor cells, known as ventral radial glia (vRG) (Haubensak et al., 2004; Lin et al., 2021; Miller et al., 2019; Tan and Shi, 2013). vRG are able to generate neurons directly, but they primarily produce intermediate progenitor cells (IPCs) and outer radial glia (oRG) that, in turn, produce neurons (Hansen et al., 2010; Miller et al., 2019). IPCs and oRG move out of the ventricular zone and constitute an expanded secondary proliferative region: the subventricular zone (SVZ). The outer SVZ (oSVZ) is the predominant source of neurons for the upper cortical layers (Nowakowski et al., 2017, 2016). Besides oRG, additional subtypes have been described in the human developing cortex, including truncated radial glia (tRG) that emerge at later stages of development and have fibers terminating abruptly in the outer SVZ (Nowakowski et al., 2016). These tRG are believed to guide neuronal migration continually, despite the loss of direct contact with the pia at later stages of neurogenesis.

### Intermediate progenitor expansion and differentiation

Around GW7, vRG cells that divide asymmetrically produce progeny that adopt neuronal fates directly, or re-enter the cell cycle as IPCs (Bystron et al., 2008; Haubensak et al., 2004; Noctor et al., 2004). IPCs are multipolar cells, primarily located in the SVZ, that do not appear to maintain contact with either the ventricular or pial surfaces. In fact, they were called intermediate progenitors for their lineage position: intermediate between RG and postmitotic projection neurons (Noctor et al., 2004). IPCs can be considered a cortex-specific form of a transit amplifying cell that can proliferate and then differentiate (Tajbakhsh and Gonzalez, 2009). These cells are not static, but extend and retract multiple processes, and can generate small numbers of glutamatergic neurons for all cortical layers (Haubensak et al., 2004; Hevner, 2019; Mihalas and Hevner, 2017; Molnar and Pollen, 2014). IPCs contribute to radial expansion and characteristic folding of the human brain (known as gyrification). These transit amplifying cells specifically express TBR2 (T-box brain protein 2), a T-box transcription factor (Englund et al., 2005; Mihalas and Hevner, 2017). Humans with deficient TBR2 expression have severe cortical malformations, characterized by microcephaly and polymicrogyria (Baala et al., 2007), highlighting the crucial requirement for IPCs in cortical development. Distinct subpopulations may also exist within the IPC population or between IPCs that divide only once, and transient amplifying progenitors that divide multiple times, but these subpopulations have not been identified to date (Miller et al., 2019; Betizeau et al., 2013). It is also conceivable that mitochondrial form and function could be essential for the ability of these progenitor cells to differentiate into cortical neurons. Studies in mice suggest that these cells may have acquired a more fragmented mitochondrial network and decreased dependence on glycolysis (Box 1) (Iwata et al., 2020; Khacho et al., 2016); a more complete characterization in human models is needed.

**Box 1. Types of metabolic respiration**

The inner mitochondrial membrane and cristae junctions house the large protein complexes of the electron transport chain (ETC) that convert NADH molecules into ATP. This process, termed oxidative phosphorylation (OXPHOS), leads to the bulk of cellular ATP production and is essential for meeting the bioenergetic needs of the cell (Chandel, 2015, 2014; Picard et al., 2016; Wallace, 2018). In the mitochondrial matrix, most of the mitochondrial metabolic activity occurs through the tricarboxylic acid (TCA) cycle, which generates three NADH and one FADH_2_ molecule. NADH and FADH_2_ can then feed the ETC complexes for ATP generation. The TCA cycle intermediates provide building blocks for macromolecules such as fatty acids, nucleotides, hemes and porphyrins. Thus, mitochondrial homeostasis influences not only energy production, but also the biosynthesis of these essential intermediates that are essential for cell survival. In general, a more fused mitochondrial network supports OXPHOS metabolism and a more fragmented mitochondrial network shifts the energetic state toward glycolysis, as seen during reprogramming. Many stem cells rely on glycolysis and have a fragmented mitochondrial network that fuses during neuronal differentiation and supports a transition to OXPHOS. Mutations in various genes involved in mitochondrial respiration have been described. These genetic dysregulations have been associated with severe neurodevelopmental disorders (Baertling et al., 2014; Inak et al., 2019; Lake et al., 2016; Romero-Morales et al., 2020; Winanto et al., 2020).

The reduction of oxygen by the electron transport chain leads to reactive oxygen species (ROS) superoxide byproducts, the accumulation of which can be damaging to cellular processes. During homeostatic conditions, ROS can also modulate metabolism by redox signaling and post-translational modifications, such as acetylation and thiol oxidation.

### Expansion and maturation of cortical layers

The cortical plate develops into the layered cerebral cortex, in which each layer contains a characteristic group of neuronal subtypes. This variety of pyramidal neuron subtypes is determined mainly by the laminar position of their cell bodies, molecular and electrophysiological properties, somatic and dendritic morphology, and axonal connectivity (Arlotta and Paşca, 2019; Lin et al., 2021; Tan and Shi, 2013). Despite the variety of pyramidal neuron subtypes there is not a consensus about the specific regulation of mitochondrial homeostasis in these essential cells of the cortex. How specific mutations impacting mitochondrial dynamics, motility or mitophagy affect the activity of pyramidal neurons at specific cortical layers is largely unknown. Pyramidal neuron activity is primarily modulated by a large class of inhibitory interneurons that migrate from the ventral forebrain to connect with pyramidal neurons and form the local cortical microcircuit (Kepecs and Fishell, 2014; Paredes et al., 2016; Wonders and Anderson, 2006). The migration of interneurons and their integration into the cortical circuit involves high energy demand processes that likely require the coordination of the dynamic properties of the mitochondria to supply the ATP and metabolites needed (Box 1), as well as to mediate signaling cascades required for optimal cellular function and quality control (Lin-Hendel et al., 2016). This high demand for mitochondrial respiratory activity is accompanied by the risk of oxidative stress due to increased electron leak from the mitochondrial respiratory chain (Box 1), which can impair mitochondrial homeostasis (Schieber and Chandel, 2014). This small leakage (estimated to be less than 0.1%) is caused by electrons from NADH or FADH_2_ that are not transferred to complex IV and by oxygen (Chandel, 2015). These electrons can react in a non-enzymatic manner with oxygen to generate superoxide. Thus, the fine balance of mitochondrial fusion and fission supports respiration and signaling events, such as Ca^2+^ oscillations, which are crucial for neuronal function and connectivity (Box 2).

**Box 2. Calcium signaling**

Calcium is considered an essential messenger capable of transferring signals within the cell as a direct response to membrane depolarization, regulating activity-dependent signaling. Calcium relays information on neuronal activity locally (e.g. in a dendritic spine) and globally to initiate switches in metabolism. Calcium homeostasis is maintained through the coordinated interaction of the endoplasmic reticulum (ER) and the mitochondria (Csordás et al., 2018). Despite the importance of these mitochondria-ER contact sites to fundamental cellular functions and the potential link to disease (Csordás et al., 2018; Vallese et al., 2020), their regulation and function in cortical development remain untested.

After the neurogenic stages described above are complete, the radial glia detach from the apical surface and become gliogenic, generating astrocytes and oligodendrocytes, or transform into ependymal cells (Kriegstein and Alvarez-Buylla, 2009). In addition to the diverse cell composition, the cortex is characterized by highly tuned expansion of layers (Bystron et al., 2008; Rakic, 1988). The variations in cellular composition and laminar proportions reflect the complex functions that these cortical areas execute during development and beyond. They also are a remarkable example of the ability of a cell to regulate proliferation and differentiation, to migrate to correct laminae, and to integrate and assemble into circuits. These coordinated events also highlight the extraordinary energy demands, metabolic adaptation and exquisite quality control, all of which rely on the fitness of the mitochondrial network.

## Mitochondrial dynamics

**Fig. 2.**
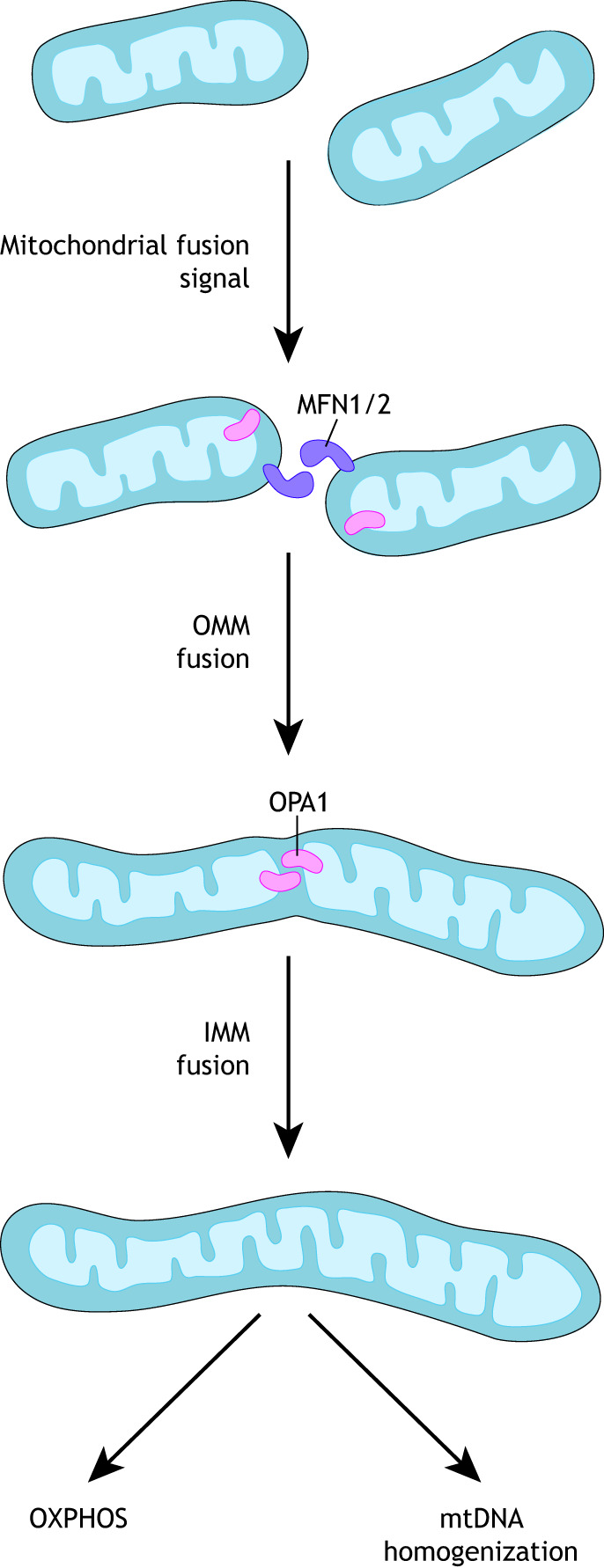
**Mitochondrial fusion.** Mitochondrial fusion occurs in two distinct steps: first, outer mitochondrial membranes fuse via mitofusin 1 and mitofusin 2 interactions (MFN1/2). Second, inner mitochondrial membranes fuse via the action of optic atrophy 1 (OPA1). Fusion helps support mitochondrial DNA (mtDNA) homogenization and supports a shift to oxidative phosphorylation (OXPHOS).

**Fig. 3.**
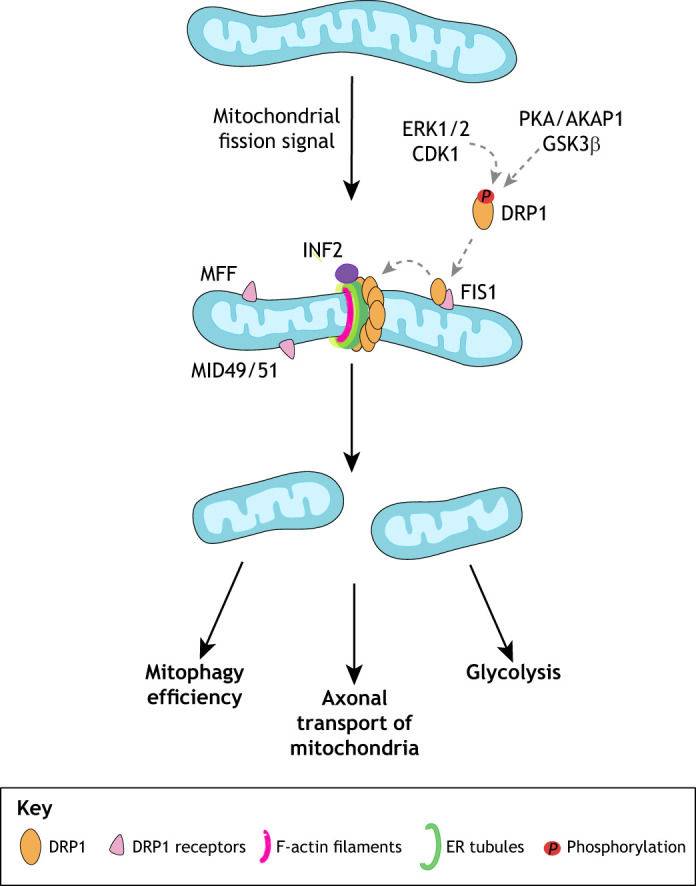
**Mitochondrial fission.** Dynamin-related protein 1 (DRP1) is the main executioner of mitochondrial fission through its oligomerization and ring formation around the outer mitochondrial membrane (OMM). A multitude of other players help regulate this process, including multiple receptor proteins on the OMM, such as MFF, FIS1 and MID49/51, which help to stabilize DRP1 recruitment. Fission occurs at sites of contact with mitochondria, F-actin and the endoplasmic reticulum (ER) with help from Golgi-derived vesicles (not pictured) that help orchestrate the complete scission of the membrane. AKAP1, A-kinase anchor protein 1; CDK1, cyclin-dependent kinase 1; ERK1/2, extracellular signal-regulated kinases 1 and 2; FIS1, mitochondrial fission 1 protein; GSK3β, glycogen synthase kinase 3β; INF2, inverted formin 2; MID, mitochondrial dynamics protein; MFF, mitochondrial fission factor; PKA, protein kinase A.

Mitochondria are major signaling organelles that undergo constant dynamic morphological changes, in flux between fragmented and fused states, in response to the energy demands of the cell (Figs 2 and 3). The dynamic properties of the mitochondria include fusion (joining of adjacent organelles), fission (fragmentation of the network), motility (transport and movement within the cell) and mitophagy (degradation) (Mishra and Chan, 2016). These mitochondrial properties are crucial for proper function. It has been over 100 years since the ability of mitochondria to fuse and divide was characterized (Lewis and Lewis, 1914); however, basic science texts continue to depict mitochondria as separate from any network and existing as static organelles. Breakthroughs in high- and super-resolution imaging techniques have given even greater insight into the truly dynamic nature of these organelles, revealing specialized network morphologies and properties based on specific cell types (Chan, 2007, 2019; Westermann, 2010; Dorn, 2013; Johnson et al., 1981; Bereiter-Hahn and Vöth, 1994; Rizzuto et al., 1996). Large dynamin-related GTPases regulate mitochondrial dynamics by inducing mitochondrial fission and fusion in a highly conserved manner (Chan, 2012; Hoppins et al., 2007; Praefcke and McMahon, 2004). It has become evident that the spatial and temporal orchestration of mitochondrial dynamics is essential within the complex morphology of neural cells and throughout stages of development (Berthet et al., 2014; Ishihara et al., 2009). A study of neural stem cells in murine cortex (Khacho et al., 2016) found that promoting mitochondrial fusion increases the ability of neural stem cells to self-renew while promoting mitochondrial fission induces differentiation. A follow-up study, which tracked the mitochondrial dynamics of neural progenitors through early stages of neurogenesis (Iwata et al., 2020), revealed that the state of the mitochondrial network (fused or fissed) determines the fate of the cells and that this decision occurs within hours of mitosis. Thus, these studies highlight the crucial role that mitochondrial morphology plays in NSC decisions and the potential involvement of mitochondrial morphology and dynamics in neurodevelopmental disorders.

### Mitochondrial fusion

Mitochondrial fusion is necessary for mitochondrial DNA (mtDNA) homogenization and tissue maturation, as well as for the formation and assembly of the electron transport chain (ETC) (Box 1) (Chan, 2012). Unlike fission, fusion of the outer and inner mitochondrial membranes comprises two distinct mechanisms (Fig. 2). Mitofusin 1 (MFN1) and mitofusin 2 (MFN2) facilitate outer membrane fusion, while optic atrophy 1 (OPA1) mediates inner membrane fusion (Alexander et al., 2000; Delettre et al., 2000; Chen et al., 2003). MFN1 and MFN2 are localized to the OMM, where they work by homo- or hetero-dimerizing with MFNs on adjacent mitochondria effectively joining fragmented mitochondria. Both MFN1 and MFN2 levels vary in expression among different tissues (Santel et al., 2003), with the brain having higher levels of MFN2 than MFN1 (Eura et al., 2003), indicating a non-redundant requirement for the mitofusins during early development (Schrepfer and Scorrano, 2016).

Fusion of the inner membrane begins through the action of OPA1. OPA1 exists as two proteolytically cleaved proteins, designated as long OPA1 (OPA1-L) and short OPA1 (OPA1-S) (Mishra et al., 2014; Del Dotto et al., 2017). OPA1-L is anchored in the IMM with its GTPase domain exposed to the intermembrane space. It coordinates the active process of IMM fusion by forming homodimers with OPA1-L proteins on the opposite target membrane. Studies in murine NSCs have revealed dramatic changes in mitochondrial morphology as NSCs commit to a neural fate and differentiate into neurons, including parallel coordinated transcriptional changes to support this metabolic shift (Beckervordersandforth et al., 2017; Khacho et al., 2016; Fame et al., 2019). For example, studies in mice indicate that enhanced mitochondrial fusion promotes NSC self-renewal through increased Notch signaling (Khacho et al., 2016; Steib et al., 2014). Conversely, transition of these NSCs to committed progenitors results in mitochondrial fragmentation (discussed below) with increased reactive oxygen species (ROS) levels (Box 1), thereby inhibiting self-renewal genes and promoting further differentiation (Khacho et al., 2016). Studies in human organoids have shown that, during cortical development, neural progenitor cells (cells expressing PAX6 and SOX2) have a more fused mitochondrial network that fragments as cells commit to TBR2-expressing intermediate progenitors and early born neurons (Romero-Morales et al., 2020). Mature neurons show a complex mitochondrial phenotype, with a more elongated network in the soma and fragmented mitochondria in axons. More systematic analysis of the morphology and dynamics of the mitochondrial network is necessary; changes in mitochondrial morphology during corticogenesis have physiological implications for stem cell commitment and metabolic adaptations. Elucidating these complex mechanisms in human model systems has tremendous implications for the basic understanding of cortical development, and for neurodevelopmental and neurodegenerative diseases.

Disproportionate levels of either mitochondrial fusion or fission results in profound pathological abnormalities, as well as neurodegenerative diseases such as Charcot-Marie-Tooth syndrome and dominant optic atrophy caused by mutations in *MFN2* and *OPA1*, respectively (Züchner et al., 2004; Alavi et al., 2007; Davies et al., 2007; Waterham et al., 2007). Although the majority of individuals with OPA1-related diseases have symptomatic optic neuropathy, other rare associations with spastic paraplegia (Carelli et al., 2015; Verny et al., 2008; Yu-Wai-Man et al., 2010), and syndromic parkinsonism and dementia (Carelli et al., 2015), reported severe defects in early neurogenesis due to depletion of neural progenitor cells or cortical specification (Inak et al., 2019; Romero-Morales et al., 2020) suggest that OPA1 function is essential in many areas of the central nervous system, particularly the cortex.

In the context of neural development, mitochondrial fusion appears necessary for the movement of mitochondria within neurons of the cerebellum (Chen et al., 2016) and for the increase of mitochondrial membrane potential (Dickey and Strack, 2011). As mentioned previously, fusion appears to improve the efficiency of OXPHOS, as well as the ability of neurons to maintain ATP levels in response to hypoxia (Chen et al., 2007; Khacho et al., 2016; Mishra and Chan, 2016; Schrepfer and Scorrano, 2016; Westermann, 2012). The correlation between mitochondrial fusion and mitochondrial membrane potential may be important for maintaining cytosolic Ca^2+^ levels with important functional consequences for dendritic spine formation and dendritic arborization (Flippo and Strack, 2017). Biochemical, cellular and state-of-the-art imaging approaches could provide new insight into the mechanisms and regulation of mitochondrial fusion during human cortical development.

### Mitochondrial fission

Dynamin-related protein 1 (DRP1) executes fission upon activation by several post-translational modifications, including phosphorylation, ubiquitylation, sumoylation and O-GlcNAcylation. The enzymes that perform this activation of dynamin-related proteins have not been completely identified, but extracellular signal-regulated kinases 1 and 2 (ERK1 and ERK2) and cyclin dependent kinase 1 (CDK1) phosphorylate DRP1 at serine 616. Additional phosphorylation sites at serines 600, 637, 693 and 656 by calmodulin-dependent protein kinase 1α (CaM kinase Iα), protein kinase A (PKA), glycogen synthase kinase 3β (GSK3β) and A-kinase anchor protein 1 (AKAP1) have been identified – among others – providing insight into specific pathway that promote increased mitochondrial fragmentation (Taguchi et al., 2007; Prieto et al., 2016; Han et al., 2008; Chang and Blackstone, 2010; Chou et al., 2012). Once activated, DRP1 translocates from the cytosol to the OMM, where it binds to receptors such as mitochondrial fission 1 (FIS1), mitochondrial fission factor (MFF) or mitochondrial dynamics protein 49/51 (MID49/MID51), and hydrolyzes GTP. DRP1 self-assembles around the mitochondria, constricting both membranes until the organelle is divided in two (Hoppins et al., 2007; Pon, 2013; Antonny et al., 2016; Francy et al., 2017) (Fig. 3).

The structural domains and mechanistic details of action for DRP1 are described elsewhere (Hoppins et al., 2007; Meglei and McQuibban, 2009; Chappie et al., 2010; Gao et al., 2010; van der Bliek and Payne, 2010; Mears et al., 2011; Francy et al., 2017). Importantly, however, structural analysis of DRP1 has revealed that the rings formed by DRP1 oligomers are smaller in diameter (30-50 nm) than the average diameter of mitochondria (Ingerman et al., 2005; Mears et al., 2011), suggesting that other constriction mechanisms are involved. The ER and the actin cytoskeleton are considered key players in mitochondrial dynamics (Lewis et al., 2016; Rehklau et al., 2017; Steffen and Koehler, 2018); ER tubules are found at sites of fission, where they form contacts with the mitochondria through the ER-mitochondria encounter structure (ERMES) complex and initiate fission even before DRP1 constriction (Elgass et al., 2015; Lewis et al., 2016; Yang and Svitkina, 2019). Tethering of the ER to mitochondria might serve not only to facilitate mitochondrial fission, but also to regulate mitochondrial membrane biosynthesis, Ca^2+^ signaling (Box 1) and protein import (reviewed by Kornmann and Walter, 2010). Following ER tubule encirclement of the mitochondria, it has been proposed that INF2-induced actin polymerization occurs, triggering an initial constriction mechanism to facilitate DRP1 recruitment, assembly and final scission of the membrane (Korobova et al., 2013; Pon, 2013). A subset of DRP1 is responsible for tubulating the ER, independent of its GTPase or oligomerization activities, and is mediated by the less-well characterized variable domain, indicating DRP1 may have fission-independent functions that have yet to be established (Adachi et al., 2020). In addition to these mechanisms, Golgi-derived vesicles driven by the activity of the small guanosine triphosphatase ADP-ribosylation factor 1 (Arf1) and or its effector PI(4)KIIIβ are required for complete fission of the mitochondria (Nagashima et al., 2020). Cryo-electron microscopy analysis has shown that GTP hydrolysis following linear filament co-assembly of DRP1 allows DRP1 to dissociate from its receptors to undergo filament shortening and curling to form its constrictive ring (Kalia et al., 2018). Moving forward, additional studies on the mechanisms by which mitochondrial fission is regulated across different cell types and cell states in human development are needed to better elucidate the intricate events coordinating mitochondrial fragmentation.

Studies of brain development in DRP1-deficient mice have revealed that the forebrain is significantly smaller than in control mice and that developmental apoptosis in the neural tube is reduced (Lewis et al., 2018). In addition, there is a significant increase in TUNEL-positive neural cells mainly in the deep cortical layers. These neurons are likely to be early-born cells that had started the final maturation process of extending neurites and forming synapses (Ishihara et al., 2009). The mechanisms by which mitochondrial fragmentation mediates all these essential cellular functions during corticogenesis have not been completely elucidated. Moreover, the impact of abnormal fission on the maintenance of human neural progenitor pools, on the ability of these progenitors to differentiate or migrate to the cortical plate, and on the function of mature neurons and glial cells is unknown.

A better understanding of the crucial function of DRP1 in cortical development may come from the analysis of clinical phenotypes in individuals with mutations in *DNM1L* (the gene for DRP1) (Table 1). The clinical presentation of individuals with pathogenic DNM1L variants, is characterized by hypotonia and developmental delay, but varies between genotypes (Whitley et al., 2018). Individuals with mutations in the GTPase domain (catalytic domain) of DRP1 show microcephaly and optic atrophy phenotypes, while individuals with mutations in the stalk domain (responsible for protein-protein interactions/folding) tend to exhibit intractable seizures (Ryan et al., 2018; Whitley et al., 2018; Yu-Wai-Man et al., 2010). With growing whole-exome sequencing efforts, more individuals are being identified with DNM1L variants, as well as variants in other dynamic machinery, such as receptor proteins Mff and MID49 (Table 1) (Bartsakoulia et al., 2018; Koch et al., 2016; Shamseldin et al., 2012). Understanding how mutations in different functional domains of DRP1 and its receptor proteins can affect neural differentiation and development is urgently needed for the treatment of these often fatal diseases. Furthermore, it would be beneficial to study the effects of these mutations on neuronal differentiation and maturation in the human cortex.

Although evidence for the function of mitochondrial fission during homeostatic cellular conditions is beginning to emerge (Kamerkar et al., 2018), the impact of mitochondrial fission in early human neurogenesis remains poorly understood. In the context of neural development, fission has been proposed to be essential for many cellular processes. First, it is plausible that generation of smaller organelles facilitates the transport of mitochondria along cytoskeletal tracks in cells – e.g. in neurons with long and thin axonal processes (Kaasik, 2015). Second, mitochondrial fragmentation is associated with increased quality control to maintain a healthy population of organelles during cell division. Third, maintenance of small mitochondrial size in central nervous system axons through MFF-dependent fission is crucial to limit presynaptic Ca^2+^ dynamics (Box 2), neurotransmitter release and terminal axonal branching (Lewis et al., 2018). Finally, smaller mitochondria allow for more-efficient engulfment by the mitophagy machinery (Burman et al., 2017).

### Cristae dynamics

Normal cristae are dynamic bioenergetic compartments formed by invaginations of the mitochondrial inner membrane to increase surface area for ETC function (Box 1). Cristae that protrude out into the matrix and form narrow openings at their base are known as crista junctions (Rehklau et al., 2017; Schorr and van der Laan, 2018; van der Laan et al., 2016) The mitochondrial contact site and cristae organizing system (MICOS complex) forms the structural basis for crista junctions and it is the major multiprotein complex regulating cristae biogenesis (Cogliati et al., 2016; Giacomello et al., 2020). The MICOS complex allows for the formation of micro-compartments known as intracristal spaces that limit the diffusion of small molecules, ATP and cytochrome c (van der Laan et al., 2016).

The process of cristae dynamics and remodeling modulates the reaction kinetics of the citric acid cycle and OXPHOS. In addition, during apoptosis, cristae dynamics facilitate the release of cytochrome c (Cogliati et al., 2013; Lizana et al., 2008). Some components of the MICOS complex have been found to be mutated in humans. For example, mutation of an essential splice site in the *C19orf70* gene (*MICOS13*) that encodes QIL1, a mitochondrial protein and component of the MICOS complex, induces severe mitochondrial encephalopathy, hepatopathy and lactate acidosis (Table 1) (Gödiker et al., 2018). Other clinical presentations included cerebellar hemisphere and vermis atrophy, Barth syndrome and optic atrophy (Eramo et al., 2020). OPA-1S is proposed to provide a more passive structural role at the mitochondrial matrix in cristae formation and maintenance (Del Dotto et al., 2018; Giacomello et al., 2020). OPA1-dependent cristae remodeling is fundamental for OXPHOS to function *in vivo* (Cogliati et al., 2013; Varanita et al., 2015). Uncovering the exact modulation of the MICOS complexes and of cristae morphology and dynamics in neural cells (neurons and glial cells), and how this regulation influences neurodevelopment, would provide additional insight into the mechanisms by which mitochondrial cristae contribute to cortical development.

## Mitochondrial motility

The unique morphology of neurons, which includes the long extension of fine processes in cortical network formation, gives rise to unique adaptations to ensure respiratory fitness throughout the cell. The machinery that governs transport throughout this specialized system has been characterized in many excellent reviews (Melkov and Abdu, 2018; Misgeld and Schwarz, 2017; Sheng and Cai, 2012). Here, we provide a closer look into the ways mitochondrial dynamics specifically support these unique bioenergetic demands and why this system may be particularly susceptible to perturbations in mitochondrial function during development.

**Fig. 4.**
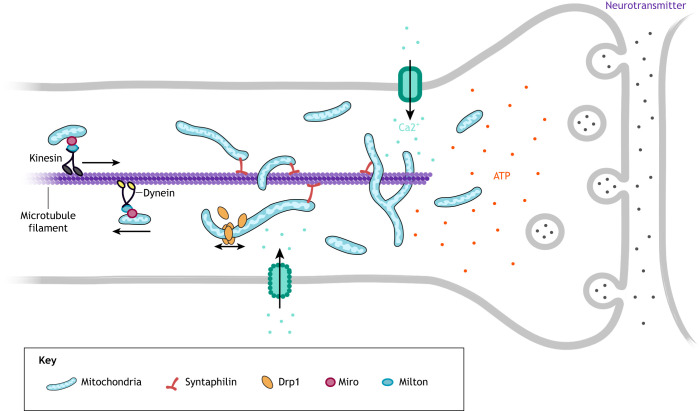
**Mitochondrial motility.** Efficient mitochondrial motility is essential to reach areas of high energy demand in cells of the central nervous system, such as at the synapse in neurons. Mitochondria are transported along microtubules with the aid of kinesin motor proteins and Miro/Milton complexes to anchor mitochondria for transport. Mitochondrial dynamics are closely tied to this process, as smaller mitochondria are more motile. Once at the synapse, mitochondria that have undergone fission can fuse with a larger local network, where they are essential for regulating ATP (red) and Ca^2+^ (teal) during neurotransmitter release (purple).

Neurons and other neural cells leverage mitochondria dynamics to fine tune their respiratory needs for distinct morphological and physiological microenvironments. Mitochondria are found in mobile and stationary pools in neurons, where a complex trafficking system ensures viable mitochondria reach sites of activity far from the somal body (Berthet et al., 2014; Mandal and Drerup, 2019; Morris and Hollenbeck, 1993; Sui et al., 2019) (Fig. 4). Two Rho GTPases of mitochondria, Miro-1 and Miro-2, regulate mitochondrial motility and transport (Chen and Chan, 2009; Chen and Sheng, 2013). The Miro/Milton complex is known to link mitochondria with kinesin proteins that are responsible for anterograde transport in neurons. Once at the inter-bouton, mitochondria are able to become stationary with the help of syntaphilin docking proteins (Kang et al., 2008) (Fig. 4). Retrograde transport, mediated by dynein motor proteins, is crucial for the return of damaged mitochondria back to the soma for degradation and anterograde transport acts to help replenish stable pools. Motile mitochondria fuse with stationary mitochondria in order to turnover aged or damaged mitochondria, and maintain the health of these pools at synaptic sites (Cheng and Sheng, 2020; Drabik et al., 2016; Lin et al., 2017; Yu et al., 2016). In fully differentiated cultured cortical neurons, mitochondria are concentrated pre- and postsynaptically where there is high energy demand and Ca^2+^ regulation (Boxes 1 and 2) (Chang et al., 2006; Son and Han, 2018; Stiles and Jernigan, 2010). Thus, if mitochondria are unable to reach the signal exchanging center during development and even after development, neuronal function will be impaired.

Motility is increased during early stages of neuronal development to meet the needs of a cell undergoing constant remodeling of synapses (Chang and Reynolds, 2006; Faits et al., 2016). At this time, mitochondria are more likely to localize near axonal branch points, as axons and dendrites undergo phases of extension and retraction during arborization (Faits et al., 2016). As maturation progresses, transport decreases to provide consistent support to stable synapses and a steady state of Ca^2+^ buffering (Box 2) and ATP production at these sites. Mature neurons have more fixed spatiotemporal requirements, allowing stable pools of mitochondria to develop and mitochondrial transport to prioritize maintaining the health of these respiratory niches (Chang and Reynolds, 2006; Cheng and Sheng, 2020). Stress signals or disease states in mature neurons may restore this mobility back to developmental levels but the specific mechanisms of compensatory increases in motility need further study (Faits et al., 2016). The LKB1-NUAK1 kinase pathway regulates axon branching by promoting mitochondria immobilization (Courchet et al., 2013). The LKB1-NUAK1 pathway is necessary and sufficient to immobilize mitochondria specifically at nascent presynaptic sites, highlighting the ability of immobile mitochondria to stimulate axon branching, a property not shared by mobile mitochondria (Courchet et al., 2013). Whether this mitochondrial regulatory system is shared by developing or mature neurons in the cortex is not known.

Neural mitochondrial dynamics impairment affects mitochondrial distribution in the axon with a progressive loss of density inversely proportional to length (Smith and Gallo, 2018; Trevisan et al., 2018; Yu et al., 2016). Cultured mouse *Drp1*^−/−^ Purkinje cells have shorter dendrites with decreasing numbers of mitochondria along the length of the process, indicating that fission machinery is required for dendritic delivery and constant outgrowth of these processes (Fukumitsu et al., 2016). Loss of this machinery results in decreased and slower transport, particularly affecting axons located furthest from the cell body (Berthet et al., 2014). Likewise, fusion machineries are implicated in developmental defects; *Opa1*^−/−^ *Drosophila* motor neurons exhibit changes in global mitochondria distribution and are significantly shorter (Yu et al., 2016). High levels of transport regulation between dendrites and axons have been shown through actin-based motors, which act as size selection filters that limit the size of mitochondria being transported away from the cell body (Lewis et al., 2018; Trevisan et al., 2018). Mitochondria are size-filtered by Mff during transport to the axon and dendrites to avoid Ca^2+^ over buffering (Box 2), and to preserve appropriate levels of neurotransmitter release in developing presynaptic terminals (Lewis et al., 2018). These studies support the notion that mitochondrial dynamics are highly regulated and finely balanced in developing neurons, acting upstream of outgrowth and synapse formation.

Mitochondria localize to the leading process of migrating NSCs along the migratory axis in cultured neurospheres, and SVZ-derived NSCs with DRP1 suppression present migratory defects (Kim et al., 2015). Similar migratory defects from the SVZ have been implicated in multiple developmental disorders hallmarked by microcephaly, such as autism spectrum disorder (ASD) and intellectual disability (Table 1). Loss of DRP1, and therefore of smaller, highly motile mitochondria, in embryonic mice causes hypoplasia and improper neural growth (Ishihara et al., 2009; Pirozzi et al., 2018; Wakabayashi et al., 2009). Loss of DRP1 *in vitro* and *in vivo* show varied outcomes depending on cell type and developmental stage. For example, in adult mice, *Drp1*^−/−^ postmitotic forebrain neurons exhibit progressive changes in mitochondrial morphology and distribution that result in mild functional alterations (Oettinghaus et al., 2016). One explanation is that fully mature neurons might be better equipped to ‘cope’ with progressive loss of dynamic function, unless subjected to significant demands in energy requirements, while developing cells may have greater cell type-specific sensitivity, resulting in increased apoptosis (Smith and Gallo, 2018). Altered mitochondrial function and defects in neural migration or increased cell death likely have overlapping mechanisms. However, more studies are needed to understand how loss of mitochondrial dynamics effects motility in neural progenitors, intermediate progenitors and fully differentiated neurons.

## Mitophagy

In addition to mitochondria motility, fusion and fission, mitophagy is another crucial dynamic property of the mitochondria. Mitophagy has emerged as a central process in regulating mitochondrial quality by removing damaged mitochondria from the cell (Priault et al., 2005; Wong and Holzbaur, 2014). However, the regulation of mitophagy and its associated pathways in cortical neurogenesis are still poorly understood (Fig. 1). As mitochondria have developed specific niches within the compartments of highly polarized cells, such as neurons and astrocytes, pathways of mitochondrial degradation are also regulated according to the cellular microenvironment (Evans and Holzbaur, 2020; Maday and Holzbaur, 2014). Long neuronal processes make transport back to the soma of all damaged organelles an inefficient mode of recycling, increasing the likelihood that mitochondrial turnover and biogenesis occur locally at the remote parts of the cell (Lin et al., 2017). Mitophagy is essential in post-mitotic cells that have lost their proliferative capacity. Cortical networks must find efficient ways to remove damaged mitochondria to avoid cell death and maintain neuronal health for the lifetime of an organism (Zhang, 2013; Evans and Holzbaur, 2019). Understanding mitophagy regulation may provide insights into which cells in the cortical network survive, and which are more vulnerable to apoptosis in health and disease.

There are different modes of mitophagy depending on the metabolic state and the specific cell type. Cells upkeep basal levels of mitophagy for the continuous housekeeping of protein turnover to meet typical ongoing metabolic requirements or to act in the acute clearance of mitochondria as the cell mediates increased metabolic demands in response to stress (Chu, 2011; McWilliams et al., 2018; Palikaras et al., 2018). Furthermore, some cell types require programmed mitophagy during their maturation to aid in differentiation and reprogramming, further implicating the spatial and temporal maintenance of mitophagy as crucial for proper development. Retinal ganglion cell development (Esteban-Martínez and Boya, 2018), cardiomyocyte maturation (Gong et al., 2015), erythrocyte differentiation (Mortensen et al., 2010) and somatic reprogramming (Vazquez-Martin et al., 2012) all require programmed mitophagy in this way. DRP1 is known to mediate mitochondrial fission, which acts concomitantly with the clearance of damaged mitochondria by mitophagy. It is believed that this fragmentation event is a key precursor for damaged mitochondria to be targeted to and to fit within the autophagosome during degradation (Yamashita et al., 2016). Neuronal cells may have an enhanced need for mitophagy to selectively degrade damaged mitochondria to avoid sacrificing the entire mitochondrial network and undergoing apoptosis; mutations in key mitochondrial dynamic or mitophagy genes may therefore be particularly damaging in these cell types and show increased developmental sensitivity.

**Fig. 5.**
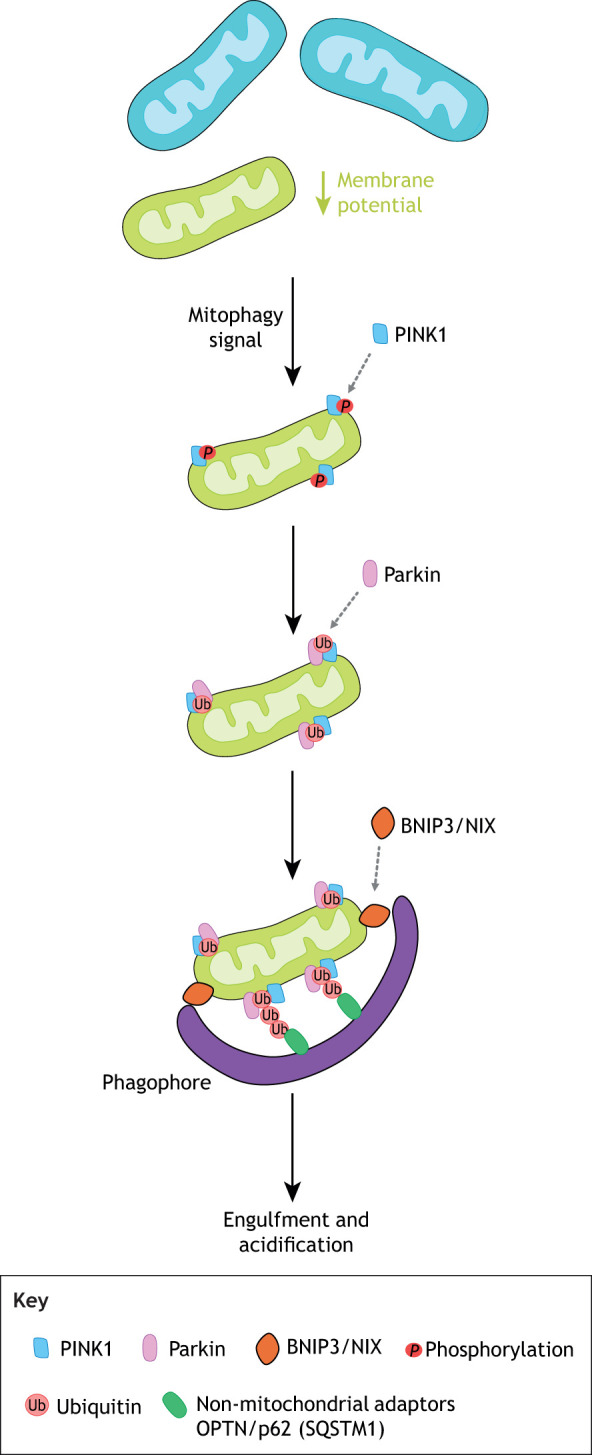
**Mitophagy.** Fragmented mitochondria are more easily targeted for mitophagy, which can be initiated via parkin-dependent and -independent pathways. There is evidence that a diverse set of adaptor and receptor molecules (including NIX/BNIP3) and non-mitochondrial adaptors, such as OPTN, p62 and SQSTM1, help regulate mitochondrial number through mitophagy; however, the outer membrane signals that target specific mitochondria for degradation are not fully understood in the context of development. BNIP3, BCL2 interacting protein 3; NIX, NIP-3-like protein X (also known as BNIP3L); OPTN, optineurin; PINK1, PTEN induced kinase 1; SQSTM1, sequestosome 1.

Most of our current understanding of mitophagy has relied on a parkin-dependent model, canonically found in stress-induced mitophagy (Fig. 5). This pathway relies on the synthesis of poly-ubiquitin chains downstream of parkin recruitment by PINK1 to the OMM (Narendra et al., 2008). Under basal conditions, mitochondrial membrane potential is maintained and PINK1 is transported to the IMM where it is degraded by PARL protease. If membrane potential is disrupted, as in the case of stress or damage, PINK1 accumulates at the OMM, where it undergoes autophosphorylation and promotes recruitment of parkin. Fully activated parkin then conjugates ubiquitin (Ub) chains to the OMM and helps to create a Ub amplification loop (Narendra et al., 2008). Autophagy-associated receptors on the OMM have emerged as regulators of the contextual specificity of mitophagy in mammals. BCL2 interacting protein 3 like (BNIP3L), which is related to the BH3-only family, and FUN14 domain-containing protein 1 (FUNDC1) are linked to mitophagy triggered by hypoxia (Hamacher-Brady et al., 2007; Liu et al., 2012; Martinez-Vicente, 2017; Zhang and Ney, 2009). Several autophagy receptors [p62, NBR1, optineurin (OPTN), NDP52 and TAX1BP1] are able to recognize polyubiquitylated signals in PINK1/parkin-mediated mitophagy (Lazarou et al., 2015; Martinez-Vicente, 2017; Vargas et al., 2019). Surface lipids, such as cardiolipin, have been shown to be necessary for the initiation of mitophagy in response to mitochondrial injury (Chu et al., 2014). These receptors communicate with non-mitochondrial adaptor proteins during the formation of the phagophore. Overall, this variety of receptors and adaptor molecules emphasizes the redundancy in mechanisms that ensure compensatory regulation of mitochondrial number (Burman et al., 2017; Evans and Holzbaur, 2020, 2019; Lazarou et al., 2015; Martinez-Vicente, 2017; Vargas et al., 2019).

There is lack of consensus about the frequency and major sites of mitophagy in neurons, likely owing to variations in cell type, model and method of stress induction. Neurons undergo robust retrograde transport, with autophagosome formation occurring at distal axons and undergoing progressive lysosomal fusion during return to the soma, but it remains unclear what percentage of mitochondria are recycled in this way (Ashrafi et al., 2014; Maday and Holzbaur, 2014). These highly polarized cells have unique adaptations to ensure efficient mitochondrial removal, such as the selective degradation of anchoring protein synaptophysin in distal axons, and degradation of mitochondrial trafficking protein Miro via PINK1/parkin under cellular stress (Lazarou et al., 2015; Lin et al., 2017; Liu et al., 2012; Wang et al., 2011). Overexpression of YFP-parkin and mitochondrial uncoupling with carbonyl cyanide m-chlorophenylhydrazone (CCCP) treatment, in mouse cortical neurons, increases retrograde transport, with parkin translocation to depolarized mitochondria predominantly in the soma and proximal dendrites (Cai et al., 2012). However, other studies using these conditions have had mixed results, arguing for enrichment in studies using more physiologically relevant perturbations, which may reveal parkin-independent pathways that occur more distally from the cell body (Miller and Sheetz, 2004; Van Laar et al., 2011; Verburg and Hollenbeck, 2008). Further adding to potential variations is the compensatory function, mitochondria-derived vesicles (MDVs) and the unfolded protein response (UPR^mt^) may have DRP1-independent approaches to removing damaged parts of the mitochondria without sacrificing the whole organelle (Gitschlag et al., 2016; Lemasters, 2014). It remains to be revealed which pathway predominates in cortical development, as well as how mitophagy is regulated in different cellular compartments and cell types throughout the developing cortex.

Mitophagy is vital for proper neurodevelopment, as dysregulated mitophagy has been found to be pathogenic in multiple syndromes and may lead to increased levels of neuroinflammation. In *OPA1*^−/−^ human primary neuronal cultures, excessive fragmentation and overactive mitophagy was found to be a possible mechanism of progressive optic atrophy in individuals with *OPA1* mutations, and mitophagy is robustly increased in Leigh/Leber hereditary optic neuropathy patient-derived fibroblasts (Dombi et al., 2016; Liao et al., 2017). Similarly, in primary neuronal cultures with loss of *WFS1* (the gene linked to causal mutations in Wolfram Syndrome), mitochondrial dynamics were impaired, axonal trafficking disturbed and neuronal development inhibited (Cagalinec et al., 2016). Pink1/parkin mitophagy was found to be overactive in these cells, with a developmental sensitivity resulting in reduction in optic nerve and brain stem volumes, which could be linked to the deficits in early brain development and pronounced psychiatric symptoms in individuals with Wolfram Syndrome (Cagalinec et al., 2016). During proinflammatory stimulation, astrocytes favored mitochondrial fission and increased mitophagy, supporting the idea that mitophagy may have a protective role in removing damaged mitochondria during neuroinflammation (Motori et al., 2013). In cases of impaired neuronal mitophagy, microglia increase activation (Lautrup et al., 2019), which may increase damaging inflammation during crucial periods of development. A few studies have highlighted specific risk genes, such as those encoding the hydrolase CRMP5 (collapsing response mediator protein 5) and the adaptor protein WDFY3 (WD repeat and FYVE domain containing 3), which may be required for differentiation and proper neuronal development via the mitophagic pathway. However, more studies are needed to profile the vast developmental genes likely linked to mitophagic dysfunction (Brot et al., 2014; Napoli et al., 2018).

## Dynamic properties of the mitochondria in glial cells

Within the human cortex there is extraordinary diversity of cell types, all of which maintain pools of mitochondria that have adapted unique motility behaviors to travel within the cell.

### Astrocytes

Astrocytes are glial cells that serve to support the function of synapses through regulating neurotransmitter uptake and recycling, as well as nutrient uptake from nearby blood vessels making them essential to neuronal survival (Allen, 2014; Allen and Barres, 2009). Astrocyte projections are interspersed throughout the brain, organized specifically to interact with thousands of neuronal synapses (Allen, 2014). A highly branched morphology with a very large surface area to volume ratio allows these vast connections. Until recently, it was thought mitochondria were too large to pass within the narrow chambers of processes as small as <50-200 nm; however, tubular mitochondria are found in the finest processes and are densely labeled in astrocyte processes along the vessel wall (Derouiche et al., 2015; Jackson and Robinson, 2015; Motori et al., 2013). High fragmentation of these mitochondria by DRP1 may be essential for their distribution. Mitochondria in astrocytes are more uniformly distributed throughout the cell than in neurons, which, in contrast, show a large pool of perinuclear clustering of mitochondria and distinct stationary pools at distal ends of dendritic and axonal processes (Stephen et al., 2015). They also have been seen to be more oscillatory and slower moving, undergoing more brief pauses and changes in direction, than in neurons (Stephen et al., 2015). It is clear these intracellular highways trafficking mitochondria differ between cell types, but the specific motor proteins or adaptor molecules regulating transport within astrocytes have not been fully studied. As in neurons, Miro1 acts as an adaptor on the OMM to anchor mitochondria to microtubule motor proteins for intracellular transport in astrocytes (Stephen et al., 2015). Mitochondrial mobility in astrocytes is regulated by neuronal activity. In rat hippocampal neurons, an increase in glutamate levels transiently decreases the mobility of mitochondria in astrocytes and reduced fusion (Derouiche et al., 2015). Such loss of mobility may help aid to create stationary pools in astrocyte axons that can support increases in synaptic activity and glutamate reuptake within the finest astrocytic processes (Derouiche et al., 2015); however, further studies are required to understand the purpose and mechanisms of these dynamics.

### Oligodendrocytes

Although mitochondrial dynamics are much less well studied in oligodendrocyte and microglial populations, there is growing interest in their function in neurodevelopment and the contribution of mitochondrial dynamics to neuronal homeostasis. Mitochondria are required for oligodendrocyte differentiation and myelination; increased Opa1 and Fis1 transcription during differentiation suggest changes in mitochondrial morphology may be important to these processes (Schoenfeld et al., 2010). Oligodendrocyte progenitor cells (OPCs) challenged with proinflammatory cytokines fail to undergo differentiation and accumulated at the progenitor stage (Bonora et al., 2014). In contrast to astrocytes and neurons, mitochondrial movement is enhanced in oligodendrocytes by increases in the excitatory neurotransmitter glutamate (Derouiche et al., 2015). It is interesting to consider that astrocytes and oligodendrocytes, although derived from the same neural progenitors, have opposing mitochondrial behavior in response to glutamate. More research is needed to understand how mitochondrial dynamics are affected by changes in neurotransmitters that help to regulate growth cones, synaptogenesis and synaptic plasticity.

### Microglia

As resident immune cells of the central nervous system, microglia are central in regulating inflammatory responses in the brain (Butovsky and Weiner, 2018), but a complete understanding of how mitochondrial dynamics regulate the function of these cells is lacking. Lipopolysaccharide (LPS)-induced stimulation of reactive microglia induces transient increases in mitochondrial fission, mediated through increases in ROS (Katoh et al., 2017; Nair et al., 2019). DRP1 suppression reduces expression of proinflammatory mediators in activated microglia, indicating that mitochondrial dynamics may help regulate microglial overactivation and subsequent neuroinflammation (Katoh et al., 2017; Nair et al., 2019; Park et al., 2013). Studies examining the damaging effects of immune response overactivation and neuroinflammation in developmental disorder liability are growing (Leffa et al., 2018; Nance et al., 2017; Rodriguez and Kern, 2011; Stolp, 2013); however, there is still much work to be done profiling mitochondria in relevant glial cell types.

## Concluding remarks and future perspectives

As described in this Review, the exact regulation of the dynamic properties of the mitochondria in the context of the human cortex remains largely unexplored. A substantial barrier to understanding the spatiotemporal mechanisms of early human neural differentiation and maturation has been the lack of a model that faithfully recapitulates human embryonic cortical development. Furthermore, the practical and ethical considerations of accessing post-mortem human embryonic tissue further adds to the challenges of studying early developmental disorders. Human iPSC-derived systems coupled with gene editing, classical biochemistry, high resolution microscopy and proteomics are rapidly evolving as a relatively high-throughput means of modeling these earliest stages of development (Arlotta and Paşca, 2019; Lodato and Arlotta, 2015; Menacho and Prigione, 2020; Velasco et al., 2019). The ability to use patient-derived samples that retain patient mutations is a powerful tool for modeling the dynamic properties of the mitochondria as well as for understanding the impact of mitochondrial dysfunction throughout cortical development.

The field of organoid biology is growing at incredible speed. Although some studies caution on the activation of cellular stress pathways that may impair cell-type specification (Bhaduri et al., 2020), extensive single cell transcriptomic and proteomic analysis support their use as models of human disease (Brennand et al., 2012; Mansour et al., 2020; Pankevich et al., 2014; Pham et al., 2018). Organoids have emerged as useful tools for increasing our understanding of mitochondrial biology in cortical development and for providing insight into the molecular details underlying neurodevelopmental disorders associated with aberrant neuronal specification, migration or maturation.

Landmark studies have shown the fundamental differences in mitochondrial morphology and function within the soma, dendrites and axons of mature cortical neurons (Berthet et al., 2014; Chang et al., 2006; Chang and Reynolds, 2006; Kaasik, 2015; Kageyama et al., 2012; Mandal and Drerup, 2019). It has also been clearly established that there are cell type-specific differences between mitochondrial form and function in neurons and astrocytes (Fecher et al., 2019; Fedorovich et al., 2017; Menacho and Prigione, 2020). However, the exact molecular regulation of mitochondrial and cristae dynamics, motility and mitophagy during the evolutionary specialization of the human cortex remains largely unexplored. Considering the rich cellular diversity of the cortex, the complex functions that are coordinated in this region of the brain and the detrimental consequences of mitochondrial dysregulation for neurodevelopment, it is essential to expand our understanding of the mitochondrial dependent signaling networks that underlie the process of human cortical development.
